# Sarcopenia: how to determine and manage

**DOI:** 10.1186/s43019-025-00265-6

**Published:** 2025-03-17

**Authors:** Jun Young Chung, Sang-Gyun Kim, Seong Hwan Kim, Cheol Hee Park

**Affiliations:** 1 Department of Orthopedic Surgery, School of Medicine, Ajou University, 164, World cup-ro, Yeongtong-gu, Suwon, Korea; 2 Department of Orthopaedic Surgery, National Medical Center, 245, Eulji-ro, Jung-gu, Seoul, South Korea; 3 Department of Orthopedic Surgery, College of Medicine, Chung-Ang University Hospital, 102, Heukseok-ro, Dongjak-gu, Seoul, Korea; 4 Department of Orthopaedic Surgery, Kyung Hee University College of Medicine, Kyung Hee University Medical Center, 26 Kyunghee-daero, Dongdaemun-gu, Seoul, Korea

**Keywords:** Muscle, Sarcopenia, Diagnosis, Prevention, Management

## Abstract

**Background:**

Understanding sarcopenia is becoming increasingly important as society ages. This comprehensive review covers the definition, epidemiology, causes, pathogenesis, diagnosis, prevention, management, and future directions for the management of sarcopenia, and the major issues related to sarcopenia in the knee joint.

**Main text:**

Sarcopenia, a condition related to aging, is characterized by decreased muscle mass and strength, which significantly affects physical function. Its prevalence may vary by region and age, with reports of up to 50% prevalence in the elderly population. The potential causes of sarcopenia include neurodegeneration, poor nutrition, changes in hormonal effects, elevated levels of proinflammatory cytokines, and reduced activation of muscle satellite cells. Various pathogeneses, such as apoptosis, proteolysis, and inhibition of the signaling for increasing muscle mass, contribute to the development of sarcopenia. Generally, the diagnostic criteria for sarcopenia are based on reduced muscle mass, reduced muscle strength, and decreased physical performance, and can be assessed using various equipment and clinical tests. A healthy lifestyle consisting of a balanced diet, sufficient protein intake, and regular exercise is recommended to prevent sarcopenia. The management of sarcopenia involves resistance exercise, proper nutrition, and deprescribing from polypharmacy. In the future, pharmacological treatment and personalized nutrition may become alternative management options for sarcopenia. Finally, since sarcopenia can be associated with knee osteoarthritis and poor outcomes after total knee arthroplasty, appropriate management of sarcopenia is important for physicians treating knee-related conditions.

**Conclusions:**

Sarcopenia is a significant pathological condition that needs to be recognized, especially in the older population. Although sarcopenia is common as aging occurs, it can be prevented by a healthy lifestyle. Currently, there are no approved drugs for sarcopenia; however, resistance exercise and proper nutritional supplementation are essential methods for managing sarcopenic conditions. Given its diverse causes, a personalized approach may be necessary to effectively manage sarcopenia. Finally, appropriate management of sarcopenia can contribute to the prevention and effective treatment of knee osteoarthritis.

## Introduction

Sarcopenia is a medical condition characterized by a progressive decrease in skeletal muscle mass and strength with age. It is closely associated with reduced physical function, disability, falls, and high mortality rates in older people. Understanding sarcopenia is becoming increasingly important as society ages. This comprehensive review covers the definition, epidemiology, causes, pathogenesis, diagnosis, prevention, management, and future directions for the management of sarcopenia, and the major issues related to sarcopenia in the knee joint.

## Definition

Sarcopenia is a term derived from the Greek word “poverty of flesh,” and it was first proposed by Rosenberg in 1989 [1]. Over the past decade, the concept of sarcopenia has evolved into “a decline in muscle mass and function with age” [2, 3]. Sarcopenia has recently been considered a progressive skeletal muscle disease, resulting in a variety of negative outcomes, such as falls, functional decline, frailty, and death [2, 4].

Controversies have led research groups to establish a clear definition of sarcopenia [2]. In 2010, the European Working Group on Sarcopenia in Older People (EWGSOP) defined sarcopenia as “a state of both reduced muscle mass and function” [5]. In the 2019 EWGSOP consensus, the definition of sarcopenia was updated to emphasize that low muscle strength is a key characteristic [6] (Table 1). It also recommends using low muscle quantity and quality to confirm the diagnosis of sarcopenia, as well as identifying poor physical performance as an indicator of severe sarcopenia (Table 1). The consensus of the Asian Working Group for Sarcopenia (AWGS), first established in 2014 and updated in 2019, defines sarcopenia as the “age-related loss of muscle mass combined with low muscle strength and/or low physical performance” [7, 8]. According to the AWGS, the age cut-off for defining “older people” is either 60 or 65 years, depending on definitions used in individual countries [7, 8].

**Table 1:** 2018 operational definition of sarcopenia in the European Working Group on Sarcopenia in Older People

Possible sarcopenia is identified based on criterion 1
A confirmed diagnosis requires the additional documentation of criterion 2
If all three criteria (1, 2, and 3) are all met, sarcopenia is considered severe
(1) Low muscle strength
(2) Low muscle quantity and quality
(3) Low physical performance

Ref. [6], the journal is open access, which permits non-commercial re-use, distribution, and reproduction in any medium, provided the original work is correctly cited

It is important to understand the concepts of “frailty” and “cachexia” to accurately define sarcopenia because the criteria for defining these conditions overlap. Sarcopenia is characterized by the loss of muscle mass and function, whereas frailty is defined as a multisystem impairment associated with increased vulnerability to stress, making it difficult to maintain homeostasis in stressful situations [9]. According to Fried’s diagnostic criteria, frailty is characterized by the presence of three or more of the following factors: unintentional weight loss, fatigue, muscle weakness, slowness, and low physical activity levels [10]. Cachexia encompasses low muscle mass and strength but is primarily driven by underlying disease-associated inflammation and metabolic disturbances, distinguishing it as a pathological subset of sarcopenia [11, 12]. This condition is usually observed in chronic diseases, such as cancer, acquired immune deficiency syndrome, or end-stage organ failure [13]. According to Evans’ criteria, cachexia is characterized by weight loss due to disease, accompanied by muscle weakness, fatigue, loss of appetite, low fat-free mass, and abnormal biochemical markers [14].

## Epidemiology

Previously, sarcopenia was not considered a disease, making it challenging to determine its prevalence. However, as the definition of this condition has become clearer, its prevalence has been reported. Previous studies using the EWGSOP definition found that 1.6% of adults aged 40–79 years in Europe [15] and 3.6% of adults aged ≥ 85 years in the UK had sarcopenia [16]. Another systematic review using the EWGSOP definition revealed that the prevalence of sarcopenia in adults aged ≥ 50 years ranged from 1% to 29% in community-dwelling populations, and increased to between 11% and 50% in those aged > 80 years [5]. Using the AWGS criteria, previous studies have shown that the overall prevalence ranges from 5.5% to 25.7%, with a higher prevalence in men (5.1–21.0%) than in women (4.1–16.3%) [17–19].

## Causes

### Neurodegeneration

With age, various parts of the neural pathway, such as the motor cortex, spinal cord, peripheral neurons, and neuromuscular junctions, undergo degeneration [20]. For instance, there was a significant decrease in the number of motor neurons, particularly those supplying fast motor units within the spinal cord. In addition, there was a loss of peripheral nerve fibers along with myelin changes. Finally, the number of neuromuscular junctions and synaptic vesicles decreases [21, 22]. These degenerative changes ultimately lead to a decrease in the number of muscle fibers and muscle mass [23, 24].

### Poor nutrition

A low protein intake can lead to a significant reduction in muscle mass and strength [25, 26]. A cross-sectional study found that almost 40% of elderly individuals aged ≥ 70 years, with an expected increase in the prevalence of sarcopenia, consume less protein than the recommended daily amount of 0.8 g/kg [27]. A nationwide population-based study with 4020 Koreans, showed that amount of protein intake and proportion of adequate protein intake (men: ≥ 55 g/day; women: ≥ 45 g/day) was significantly lower in the sarcopenia group than in the normal group [28].

In addition, vitamin D deficiency contributes to the development of sarcopenia. Vitamin D binds to receptors on muscle fibers and increases their size, thereby improving muscle strength and physical performance [29–32]. The production and metabolism of vitamin D change with age, leading to vitamin D deficiency, which is associated with sarcopenia [33]. Previous studies have shown that a low serum vitamin D level is independently related to the loss of muscle mass and decline in muscle strength in older people, which implies that older people with vitamin D deficiency are extremely susceptible to developing sarcopenia [34–36].

### Changes in hormonal effects

Hormonal changes during aging significantly affect muscle mass and strength and contribute to sarcopenia. At approximately 50 years of age, men and women have hormonal decline; men have andropause, characterized by a reduced testosterone level, while women have menopause, marked by decreased levels of estrogen, growth hormone (GH), and related factors [37, 38]. The decreased levels of these hormones and related factors are associated with loss of muscle mass and diminished capacity for muscle regeneration.

Testosterone, which is produced by the Leydig cells in men and ovarian thecal cells in women, plays a vital role in the protein synthesis and maintenance of muscles [39]. It decreases with age, starting at approximately 30 years of age, leading to gradual reductions in muscle mass and strength [39]. A low testosterone level is linked to muscle atrophy and other medical problems, including metabolic syndrome and diabetes, which further impair muscle function [40].

In women, menopause results in reduced estrogen, GH, and insulin-like growth factor 1 (IGF-1), contributing to impaired muscle performance [39]. These substances are critical for the maintenance of muscle mass and strength. In particular, IGF-1 promotes satellite cell production and contractile protein synthesis, which are essential for muscle repair and growth [39]. In addition to the menopause, aging also reduces the levels and responsiveness of GH and IGF-1, which contribute to the development of sarcopenia [39].

### Elevated proinflammatory cytokines

Chronic systemic inflammation, common in older people, plays a significant role in reducing skeletal muscle function [41]. Increased levels of proinflammatory cytokines during systemic inflammation directly cause muscle wasting by promoting the breakdown of muscle myofibrillar proteins and reducing protein synthesis [42, 43]. In particular, previous studies have reported that tumor necrosis factor-alpha (TNF-α) and interleukin-6 (IL-6) are closely linked to sarcopenia [44, 45]. TNF-α boosts ubiquitin-dependent protein degradation and triggers apoptosis, which has been associated with sarcopenia [44, 45]. IL-6 has a more pronounced catabolic role in obese individuals and contributes to sarcopenic obesity, in which lean body mass declines while fat mass is preserved or increases [45].

### Reduced activation of satellite cells

Satellite cells, known as skeletal muscle-specific somatic stem cells, play a crucial role in muscle regeneration [46]. Muscle satellite cells reside between the muscle cell membrane and the basement membrane, and are normally dormant. When a muscle fiber is damaged, satellite cells are activated, proliferate, and fuse with the muscle fibers to repair the damaged area. However, aging leads to a decline in the number of satellite cells, impairing the regenerative potential of muscle [47, 48]. In addition, senescent satellite cells have a diminished ability to proliferate, which is due to reduced expression of the Notch ligand, Delta [47–49]. These age-related changes may contribute to decreased muscle mass and strength, ultimately leading to the development of sarcopenia.

## Pathogenesis

### Apoptosis

Apoptosis is crucial for maintaining homeostasis in multicellular organisms, and is a mechanism contributing sarcopenia. Apoptosis is unique in muscle cells because of the multinucleated nature of these cells. The process is characterized by “myonuclear apoptosis,” where muscle cell nuclei condense and decrease in number without complete cell death [50].

### Proteolysis

Autophagy (self-consumption), calpain activation, and the ubiquitin–proteasome system are proteolytic mechanisms linked to sarcopenia. Autophagy enables cells to survive when nutrients are scarce by breaking down their components. Studies indicate that autophagy is the primary protein degradation mechanism in muscles under limited amino acid supply [51]. Calpain, a calcium-activated protease, does not directly break down muscle contraction-related proteins but it facilitates the degradation of sarcomere proteins through other proteolytic systems that cleave proteins attached to actin and myosin. Activation of the calpain pathway is observed during muscle atrophy due to extended inactivity [52]. The ubiquitin–proteasome system tags proteins earmarked for degradation and guides them to be broken down by a proteolytic complex. Previous studies have shown an increased expression of ubiquitin and proteolytic complexes during muscle atrophy [53].

### Akt signal and myostatin

Protein kinase B (Akt) activates the mammalian target of rapamycin (mTOR) signaling kinase, which contributes to increased skeletal muscle mass. Several studies have shown that Akt/mTOR/ribosomal protein S6 kinase (p70S6K) signaling is selectively decreased in type II muscle fibers in elderly individuals [54]. Myostatin reduces Akt/mTOR/p70S6K signaling and inhibits skeletal muscle growth. It has been reported that testosterone increases muscle mass by reducing myostatin expression [55]. The physiological phenotype of myostatin gene knockout mice also shows increased muscle mass [56].

## Diagnosis

### Guidelines to diagnose sarcopenia

In 2010, the EWGSOP consensus proposed diagnostic criteria for sarcopenia that considers both muscle mass and function [57, 58]. In 2019, the consensus was updated to include a clinical algorithm for diagnosing sarcopenia and to provide clear cutoff points for measuring variables that identify and characterize sarcopenia [6].

**Fig. 1:**
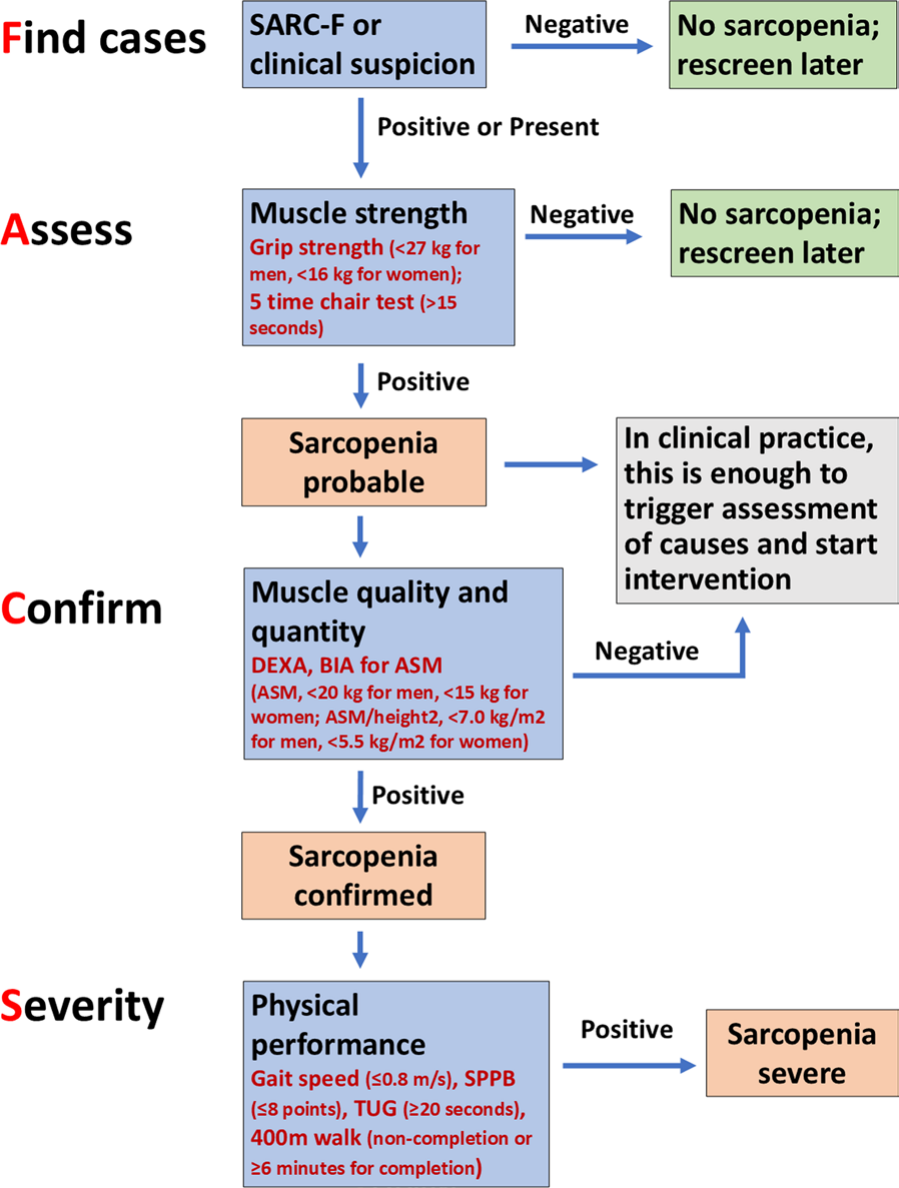
Find-Assess-Confirm-Severity (F-A-C-S) pathway in the European Working Group on Sarcopenia in Older People 22 algorithm. *SARC-F* strength, assistance in walking, rising from a chair, climbing stairs, and falls questionnaire, *DXA* dual-energy X-ray absorptiometry, *BIA* bioelectrical impedance analysis, *ASM* appendicular skeletal muscle mass, *SPPB* short physical performance battery, *TUG* timed-up and go-test. Ref. [6], the journal is open access, which permits non-commercial re-use, distribution, and reproduction in any medium, provided the original work is correctly cited

In 2019, the EWGSOP consensus recommended a four-step diagnostic pathway for sarcopenia, represented as Find cases-Assess-Confirm-Severity (F-A-C-S) (Fig. 1). First, individuals at risk for sarcopenia should be identified using the strength, assistance in walking, rising from a chair, climbing stairs, and falls (SARC-F) questionnaire, or through clinical suspicion to determine sarcopenia-associated symptoms (Finding-cases step). Second, muscle strength should be assessed using grip strength (< 27 kg for men, < 16 kg for women) or a five-time chair test (> 15 s) (assess step). Third, sarcopenia should be confirmed by detecting low muscle quantity/quality, typically evaluated using whole-body dual-energy X-ray absorptiometry (DEXA) or multifrequency bioelectrical impedance analysis (BIA) for appendicular skeletal muscle mass (ASM, < 20 kg for men, < 15 kg for women; ASM/height^2^, < 7.0 kg/m^2^ for men, < 5.5 kg/m^2^ for women) (confirming step). Finally, the severity of sarcopenia should be evaluated using physical performance measures such as gait speed (≤ 0.8 m/s), short physical performance battery (SPPB) (≤ 8 points), timed-up and go-test (TUG) (≥ 20 s), and 400 m walk test (non-completion or ≥ 6 min for completion) (severity step).

The AWGS established diagnostic criteria based on Asian data in 2014, considering that Asians exhibit different physical characteristics, cultural differences, and lifestyles than Western populations [7]. In 2019, the AWGS updated its diagnostic criteria. Although they maintained the original definition, they revised the diagnostic algorithm, protocols, and some criteria [8].

**Fig. 2:**
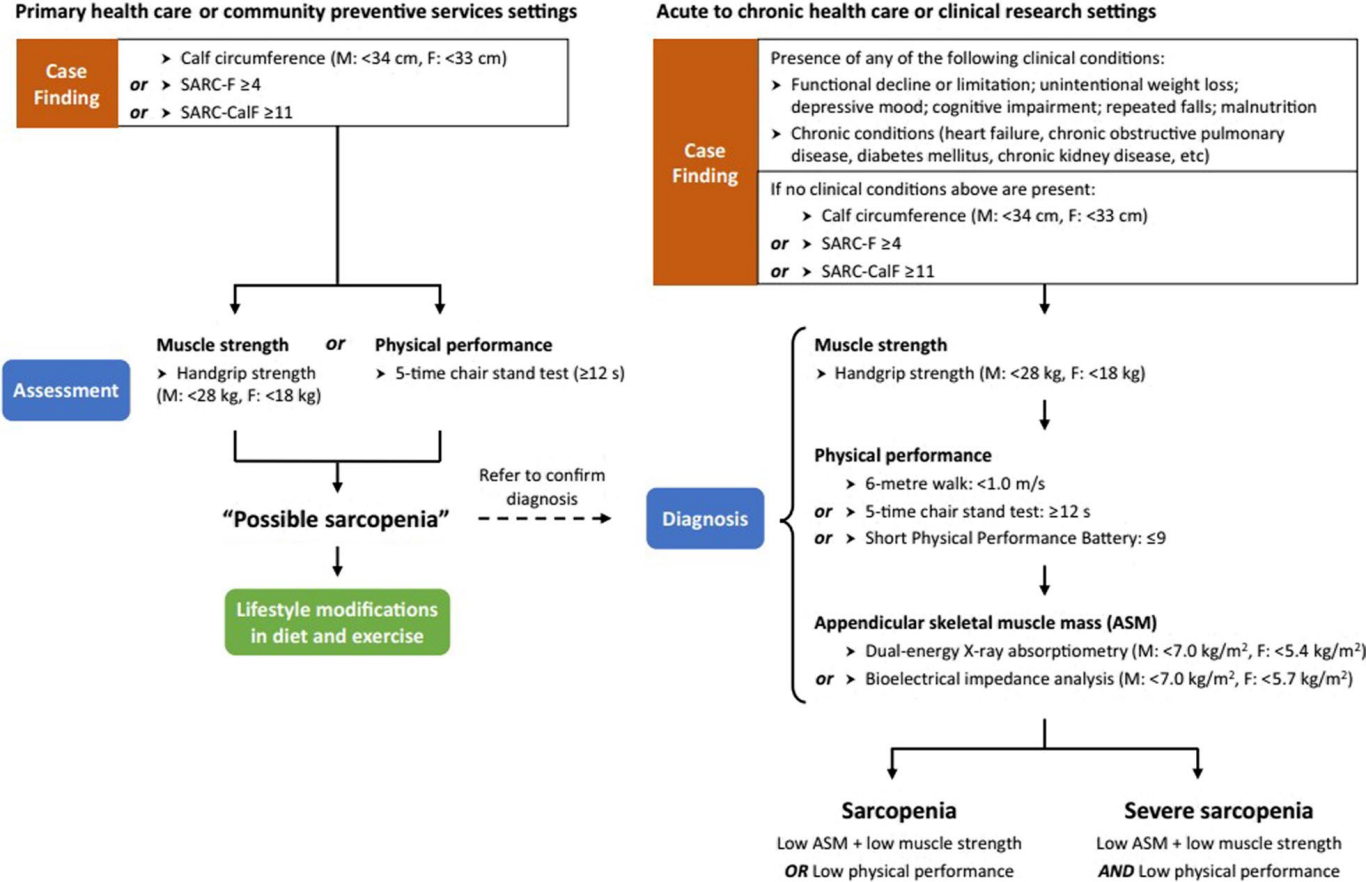
Asian Working Group for Sarcopenia 2019 algorithm for sarcopenia. Ref. [8], the authors received permission from the publisher of the journal to reuse the image

On the basis of the latest diagnostic criteria of the AWGS 2019, both muscle quality (function) and quantity should be assessed to diagnose sarcopenia. A patient is diagnosed with sarcopenia if they meet two of the following three criteria: (1) reduced muscle mass; (2) reduced muscle strength; and (3) reduced physical performance. Patients who meet all three criteria are categorized as having “severe sarcopenia” [8] (Fig. 2). To measure muscle mass, height-adjusted muscle mass (kg/m^2^) is evaluated using whole-body DEXA (< 7.0 kg/m^2^ for men, < 5.4 kg/m^2^ for women) or multifrequency BIA (< 7.0 kg/m^2^ for men, < 5.7 kg/m^2^ for women) [8, 59]. Muscle strength is measured using hand grip strength. Two types of dynamometers are used for measuring grip strength: spring-type and hydraulic-type. When using a spring-type dynamometer, the measurement is taken with the arm extended and standing. For a hydraulic-type dynamometer, the measurement is taken with the arm bent at 90° while seated [60]. The diagnostic criterion is based on the maximum value obtained from at least two grip strength measurements (< 28 kg for men, < 18 kg for women) [8, 61]. Physical performance is assessed using the SPPB (score ≤ 9), the 6 min walking test (walking speed < 1.0 m/s), or the five-time chair stand test (≥ 12 s to complete) [8, 62].

The AWGS 2019 consensus recommends additional strategies for early identification of individuals with sarcopenia, or those at risk for it, to facilitate necessary interventions (Fig. 2). First, specific criteria for case finding were suggested. The consensus recommends using either calf circumference or the SARC-F or SARC and calf circumference (SARC-CalF) questionnaires for case finding. Calf circumference should be measured as the maximum value of both calves using a non-elastic tape measure (< 34 cm for men, < 33 cm for women) [8]. Regarding SARC-F, which was also used for case finding in the 2019 EWGOSP, a score of ≥ 4 requires further assessment [8, 63]. SARC-CalF improves the sensitivity of SARC-F by adding calf circumference measurements, and a score of ≥ 11 requires further assessment. Second, the AWGS 2019 consensus introduced the new concept of “possible sarcopenia,” defined as low muscle strength with or without reduced physical performance. This concept is recommended for use in primary health care and community preventive service settings but not in hospital or research settings [8]. It can facilitate the screening of sarcopenia in places where the use of diagnostic equipment, such as BIA devices or whole-body DEXA, is challenging. If “possible sarcopenia” is diagnosed, the patient should be referred to a hospital where the use of the equipment is available to confirm sarcopenia [8, 64].

### Novel tests and tools for diagnosing sarcopenia

Computed tomography (CT)- or magnetic resonance imaging (MRI)-based diagnostic approaches for sarcopenia have been systematically evaluated in previous studies. CT imaging of the third lumbar vertebra, which correlates with the total body skeletal muscle mass, was used to detect sarcopenia and predict outcomes of sarcopenia [65–67]. Similarly, the mid-thigh muscle area, measured using MRI or CT, provides highly sensitive data on muscle changes and is strongly correlated with the total body muscle volume [66, 68, 69]. In addition, muscle quality has been assessed using MRI or CT by determining the infiltration of fat into muscle using the attenuation of the muscle [70, 71]. High-resolution imaging is expected to become increasingly common, starting in research settings and extending to clinical practice to enhance early detection of sarcopenia.

Ultrasonography is gaining traction as a reliable, portable, and cost-effective tool for measuring muscle thickness, cross-sectional area, and quality. In particular, ultrasonography can detect infiltration of fat, which is valuable for assessing muscle quality [72, 73]. While data are available for older people, more studies are required to validate the predictive ability for those with varying health conditions and functional statuses [66, 74].

The creatine dilution test is another novel method to diagnose sarcopenia, in which the total body muscle mass is estimated. The urinary creatinine level after the administration of deuterium-labeled creatine showed a strong correlation with MRI-based measurements of muscle mass [75].

Finally, the Sarcopenia Quality of Life questionnaire (SarQoL) was used to evaluate the impact of sarcopenia on the quality of life. This self-report tool addresses physical, psychological, and social dimensions, and has been validated for use in clinical and research settings [76]. The sensitivity of the SarQoL to changes in patient status over time requires validation through longitudinal studies to establish its potential as a proxy measure of treatment efficacy [66, 76, 77].

### Initiative for a global consensus on sarcopenia

Despite advances in definition and diagnostic methods, the absence of a well-established global consensus limits the efficient clinical management of sarcopenia. The Global Leadership Initiative on Sarcopenia (GLIS) aims to unify definitions and diagnostic criteria, fostering a global consensus to advance research and clinical implementation. This initiative emphasizes a standardized conceptual framework, the development of reliable diagnostic tools, and operational criteria for measuring outcomes [11, 12, 66].

In 2024, the GLIS suggested a global conceptual definition of sarcopenia through an international two-phase Delphi consensus process [78]. The six accepted general aspects of sarcopenia with strong agreements were that sarcopenia is a generalized disease of skeletal muscle (agreement, 85.4%), the prevalence of sarcopenia increases with age (98.3%), the conceptual definition of sarcopenia should not vary by the setting of care (91.2%), the conceptual definition of sarcopenia should not vary by age or condition (83.2%), the conceptual definition of sarcopenia should be the same for clinical practice and research (92.0%), and the conceptual definition of sarcopenia should be that it is a potentially reversible disease (84.1%). The three accepted components of sarcopenia with strong agreements were muscle mass (89.4%), muscle strength (93.1%), and muscle specific strength [e.g., muscle strength/muscle size (80.8%), which should all be included in the conceptual definition of sarcopenia]. The 11 accepted outcomes of sarcopenia with strong agreements were increased risk of impaired physical performance (97.9%), walking limitation (96.1%), transfer from chair or bed rising limitations (95%), falls (94.6%), fractures (89.4%), inability to perform instrumental activities of daily living (88.6%), inability to perform basic activities of daily living (self-care) (90.7%), hospitalizations (91%), new admission to care (nursing) homes (89.5%), poor quality of life (91.8%), and mortality (91.6%). A key finding of the Delphi survey was that muscle mass, muscle strength and muscle-specific strength were all accepted as “components of sarcopenia,” whereas impaired physical performance was accepted as an “outcome” rather than a “component of sarcopenia.”

## Prevention of sarcopenia

The Australian and New Zealand Society for Sarcopenia and Frailty Research (ANZSSFR) recommends adopting a healthy lifestyle to prevent sarcopenia. This involves maintaining a balanced diet, ensuring adequate protein intake, and exercising regularly [79]. Furthermore, they advise that individuals with risk factors for sarcopenia should follow personalized physical exercise and dietary plans developed by professionals.

Calvani et al. [80] suggested that for patients with sarcopenia, consuming plant-based foods such as plant protein, ample fruits, vegetables, nuts, and plant oils instead of meat and processed foods can be beneficial. This dietary approach provides balanced vitamins and ample antioxidants to the muscles, thereby improving muscle mass and strength. Liu et al. [81] conducted a prospective study to explore the effects of depression and maintaining a healthy lifestyle on the incidence of sarcopenia. The study evaluated 9486 participants from the China Health and Retirement Longitudinal Study between 2011 and 2015. They assessed lifestyle on the basis of body mass index, alcohol consumption, smoking, social activities, and sleep duration. The analysis revealed that depression is a risk factor for sarcopenia (hazard ratio = 1.34, 95% CI 1.19–1.50). Moreover, even with depression, maintaining a healthy lifestyle reduced the risk of sarcopenia by 18% compared with those who did not maintain a healthy lifestyle. The study concluded that maintaining a healthy lifestyle can reduce the risk of sarcopenia, even though this risk is higher in patients with depression.

## Management

### Resistance exercise

Resistance exercise is the most effective method for managing sarcopenia. Significant improvement in the sarcopenia-related index has been observed with resistance exercise for 3 months or longer [82].

Shen et al. [83] conducted a network meta-analysis of the effects of resistance exercise on sarcopenia. The study included 42 randomized controlled trials reported up to March 2022 and analyzed the quality of life, grip strength, and physical function (evaluated by walking speed, TUG Test, and five-time chair stand test) of 3728 patients with sarcopenia (median age: 72.9 years, 73.3% female). The study found that the quality of life of sarcopenia patients was effectively improved by resistance exercise, regardless of the presence of nutrition or aerobic and balance exercises (high- or moderate-certainty evidence). Grip strength was improved by resistance exercise combined with nutrition and balance exercises (moderate-certainty evidence). Walking speed was effectively improved by resistance exercise combined with balance exercise, regardless of nutrition (moderate-certainty evidence). The TUG test score was improved by resistance exercise combined with balance exercise regardless of nutrition (moderate-certainty evidence). The five-time chair stand test was improved by resistance exercise combined with aerobic and balance exercises, regardless of nutrition (high-certainty evidence). In conclusion, resistance exercise combined with aerobic and balance exercises improved the quality of life and physical function of patients with sarcopenia, regardless of nutrition; the inclusion of nutrition helped improve muscle strength.

Although systematic, personalized exercises administered by certified medical professionals are considered ideal for improving function in patients with sarcopenia [79], it is also possible to enhance the function of these patients through simple and easy exercises that can be performed in daily life without expert assistance.

Yoshimura et al. [84] examined whether chair stand exercises, which can be easily performed without special equipment or location restrictions, help to improve the function of stroke patients with sarcopenia. During rehabilitation, patients were encouraged to perform repetitive chair stand exercises and were evaluated for skeletal muscle index and handgrip strength at discharge. The analysis of 302 patients (mean age 78.6 years, 48.6% male) revealed that the frequency of chair stand exercises reduced the prevalence of sarcopenia at discharge (odds ratio (OR) 0.986, *p* = 0.010) and improved the muscle index and grip strength (skeletal muscle index, *β* = 0.181, *p* < 0.001; hand grip strength, *β* = 0.101, *p* = 0.032). The study concluded that performing chair stand exercises, which can be easily incorporated into daily life, can contribute to an improvement in the sarcopenic condition.

### Nutrition

Adequate protein intake, such as whey protein rich in leucine, and sufficient hydration are crucial for the nutritional management of sarcopenia [80]. The ANZSSFR recommends a daily protein intake of 1–1.5 g/kg for elderly sarcopenia patients [79]. The Korea National Health and Nutritional Examination Survey reported that insufficient hydration increases the risk of sarcopenia [85]. In addition, the intake of vitamin D, creatinine, omega-3 fatty acids, and probiotics is known to help manage sarcopenia.

Chang et al. conducted a meta-analysis to investigate the effects of whey protein, leucine, and vitamin D in patients with sarcopenia [86]. The study included three randomized controlled trials comprising a total of 637 patients and analyzed muscle mass, grip strength, and physical function in patients with sarcopenia supplemented with whey protein, leucine, and vitamins. With exercise, the nutrition-supplemented group showed significant improvements in muscle mass, grip strength, and physical function compared with the non-supplemented group. In the absence of exercise, there was no difference in grip strength and physical function between the two groups; however, muscle mass significantly improved in the nutrition-supplemented group. The study concluded that nutritional supplementation with whey protein, leucine, and vitamin D increases muscle mass in patients with sarcopenia. In addition, when combined with exercise, nutritional supplementation can improve muscle strength and physical function.

### Deprescribing from polypharmacy

Polypharmacy is also associated with the development of sarcopenia. A recent systematic review reported that sarcopenia was associated with a higher prevalence of polypharmacy [OR 1.65; 95% confidence interval (CI) 1.23–2.20; *I*^2^ 84%; *p* < 0.01] and higher number of medications (mean difference, 1.39; 95% CI 0.59–2.19; *I*^2^ 95%; *p* < 0.01) [87]. Another systematic review reported that the risk of sarcopenia was associated with polypharmacy in community-dwelling older people [88]. On the basis of these studies, it is advisable to consider deprescribing to improve the functioning of patients with sarcopenia who are on multiple medications.

Kose et al. [89] assessed the effects of deprescribing on the functional recovery and home discharge of stroke patients with sarcopenia. They focused on patients aged ≥ 65 years with sarcopenia and stroke who had been prescribed at least five medications between 2015 and 2021. The study evaluated functional independence, measured by motor activity (functional independent measure-motor), and the probability of home discharge at the time of discharge. The researchers analyzed the impact of deprescribing on these outcomes. The study encompassed 153 patients, with an average age of 81.1 years and 46.4% being male. Among them, 56 (36.6%) underwent deprescribing. The findings indicated that deprescribing had a beneficial effect on the functional independent measure-motor score at discharge (*β* 0.137; *p* = 0.017) and the likelihood of home discharge (OR 1.393; *p* = 0.002). This study, despite being conducted only on patients after a stroke, and thus having a limitation in generalizability, suggests that deprescribing from polypharmacy has the potential to improve function in patients with sarcopenia.

## The future direction of treatment

### Pharmacological treatment

Currently, no drugs have been approved by the Food and Drug Administration for treating sarcopenia. However, research on pharmacological treatments is ongoing, and some drugs are undergoing clinical trials.

A clinical trial was conducted to assess the effectiveness and safety of selective androgen receptor modulators (SARMs) for treating sarcopenia in women aged ≥ 65 years [90]. The trial involved a treatment group that received SARMs and a control group that received a placebo. All participants also received vitamin D and protein supplements. The trial included 170 patients, and their lean body mass, muscle strength, and physical function were measured. After 6 months, the treatment group showed significantly higher lean body mass than the control group (*p* < 0.001). However, there were no significant differences in muscle strength and physical function between the two groups. In conclusion, while SARMs may increase lean body mass in women with sarcopenia, they did not significantly improve muscle strength or physical function in this study.

Clinical trials have been conducted on bimagrumab, a monoclonal antibody that inhibits myostatin and activin A. In a phase II study, Rook et al. [91] reported that patients with sarcopenia who used bimagrumab showed significantly increased grip strength and thigh muscle mass compared with those who did not use the drug. In addition, the use of bimagrumab in patients with impaired function and sarcopenia improved the results of the 6 min walking test. However, in a phase III study, the efficacy of bimagrumab was not demonstrated [57]. In elderly patients with sarcopenia (average age 79.1 years) who were following an appropriate diet and exercise regimen, the use of bimagrumab demonstrated an increased lean body mass but did not show significant benefits in the SPPB and the 6 min walking test.

### Personalized nutrition supply

Different metabolic responses can occur even with the same foods because of personalized factors, such as sex, gut microbiota, and lifestyle. The Personalized Response to Dietary Composition Trial study, which analyzed postprandial metabolic responses in 1002 twins and other healthy adults, reported considerable variability among individuals in postprandial responses, such as blood triglyceride, glucose, and insulin levels, even after consuming the same meals [92]. Individual factors, such as the gut microbiota, significantly affect postprandial lipemia, and genetic factors also contribute to the variability in individual metabolic responses after meals.

Personalized nutrition could assist in more effectively preventing and managing sarcopenia, considering the different metabolic responses due to personalized factors [80]. A randomized controlled trial reported that a personalized dietary approach improved 400 m walk time and leg extension strength in 276 community-dwelling older people with lower habitual protein intake [93]. However, to date, the evidence does not seem sufficient to clearly prove the benefits of personalized nutrition for sarcopenia. More, well-designed, large-scale studies are required to obtain robust conclusions.

## Major issues related to sarcopenia in the knee joint

### Association between sarcopenia and knee osteoarthritis

Knee osteoarthritis and sarcopenia are common conditions; therefore, it is not surprising that they often coexist, raising the possibility of interaction. The biomechanical effects resulting from the altered crosstalk between bones and periarticular muscles may lead to muscle atrophy or weakness, which, in turn, can influence the onset, progression, and severity of osteoarthritis.

Pegreffi et al. [94] conducted a systematic review and meta-analysis to assess the prevalence of sarcopenia in patients with knee osteoarthritis compared with that in individuals without knee osteoarthritis. Four studies, with a total of 7495 participants [mean age: 68.4 years; predominantly female (72.4%)], were included in the analysis. The prevalence of sarcopenia in individuals with knee osteoarthritis was 45.2%, whereas it was 31.2% in the control group without osteoarthritis. The risk of sarcopenia in the knee osteoarthritis group was more than twice that of the control group (OR 2.07; 95% CI 1.43–3.00; *I*^2^ 85%).

Yang et al. [95] investigated the causal associations between knee osteoarthritis and sarcopenia using a bi-directional Mendelian randomization approach. Data from 62,497 patients with knee osteoarthritis and 333,557 healthy controls obtained from large published genome-wide association studies were analyzed. The results showed that low grip strength did not have a causal effect on knee osteoarthritis (OR = 1.205; 95% CI 0.837–1.734; *p* = 0.316), whereas appendicular lean mass, indicating musculature of the arms and legs, had a causal effect on knee osteoarthritis (OR = 1.104; 95% CI 1.041–1.171; *p* = 0.001). Conversely, knee osteoarthritis did not have a significant causal effect on grip strength or appendicular lean mass. These results from this study suggest that sarcopenia may contribute to the development of knee osteoarthritis through changes in muscle composition rather than muscle strength, although there is little evidence supporting a causal effect of osteoarthritis on sarcopenia.

On the basis of these studies, sarcopenia is considered a risk factor for the development of knee osteoarthritis, and appropriate management of sarcopenia may contribute to the prevention of osteoarthritis. However, some studies have suggested that sarcopenia itself does not affect knee osteoarthritis. Misra et al. [96] reported that obesity and sarcopenic obesity are associated with the risk of knee osteoarthritis, but they did not find an association between sarcopenia itself and knee osteoarthritis. The study implies that body weight, rather than sarcopenia itself, may be a significant risk factor for knee osteoarthritis. Considering this study, it is necessary to separately analyze patients with sarcopenia and sarcopenic obesity to clearly determine the impact of sarcopenia on osteoarthritis.

### The impact on outcomes following total knee arthroplasty (TKA)

Sarcopenia can impair recovery and increase the risk of complications in patients undergoing TKA, which is commonly performed in older patients with osteoarthritis. Appropriate management of sarcopenia contributes to the improvement of outcomes post-TKA.

He et al. [97] investigated the relationship between sarcopenia and clinical and functional outcome scores, post-TKA, in patients aged > 65 years. This case–control retrospective study evaluated the postoperative Knee Society Clinical and Function scores and perioperative complications in 180 patients with sarcopenia and 345 comparatively healthy patients, with assessments conducted at a mean of 1 year post-surgery. The results showed that patients with sarcopenia had significantly lower Knee Society Clinical (83.0 versus 88.2, *p* < 0.01) and Functional (79.2 versus 86.1, *p* < 0.01) scores, as well as higher postoperative complication rates (14.1 versus 4.1%, *p* < 0.01) compared with the healthy control group.

Sumbal et al. [98] conducted a systematic review and meta-analysis to investigate the prevalence of sarcopenia in patients undergoing TKA and its impact on medical and surgical outcomes post-TKA. Thirteen studies with 399,097 patients were included. The results showed that the pooled prevalence of sarcopenia in patients undergoing TKA was 20.1% (95% CI 13.6–28.8%; *p* < 0.00001; *I*^2^ 94.7%). Sarcopenia in patients undergoing TKA was associated with an increased risk of various complications, including blood transfusion (OR 4.68; 95% CI 3.51–6.25; *p* < 0.00001), pneumonia (OR 1.94; 95% CI 1.14–3.30; *p* = 0.01), urinary tract infection (UTI) (OR 1.64; 95% CI 1.31–2.05; *p* < 0.001), prosthetic fracture (OR 2.12; 95% CI 1.51–2.98; *p* < 0.0001), prosthetic dislocation (OR 1.99; 95% CI 1.62–2.44; *p* < 0.00001), and mechanical loosening (OR 1.78; 95% CI 1.43–2.22; *p* < 0.00001).

Sarcopenia, a dreaded complication of TKA, can also be associated with periprosthetic joint infection. Babu et al. [99] conducted a retrospective case–control study to investigate the impact of sarcopenia on periprosthetic joint infection. They compared the psoas-lumbar vertebral index, a validated marker of sarcopenia, between 30 patients with periprosthetic joint infection and 69 controls. The results showed that the mean psoas-lumbar vertebral index was significantly lower in patients with infection, and a higher index was associated with greater protection against infection (OR 0.28; 95% CI 0.109–0.715, *p* = 0.008). In conclusion, sarcopenia was identified as a risk factor of periprosthetic joint infection following total joint arthroplasty.

## Conclusions

Sarcopenia is a significant pathological condition that needs to be recognized, especially in the older population. Although sarcopenia is common as aging occurs, it can be prevented by a healthy lifestyle. Currently, there are no approved drugs for sarcopenia; however, resistance exercise and proper nutritional supplementation are essential methods for managing sarcopenic conditions. Given its diverse causes, a personalized approach may be necessary to effectively manage sarcopenia. Finally, appropriate management of sarcopenia can contribute to the prevention and effective treatment of knee joint diseases, including osteoarthritis.

## Data Availability

Not applicable.

## Abbreviations

Akt: Protein kinase B
ANZSSFR: Australian and New Zealand Society for Sarcopenia and Frailty Research
ASM: Appendicular skeletal muscle mass
AWGS: The Asian Working Group for Sarcopenia
BIA: Bioelectrical impedance analysis
CI: Confidence interval
CT: Computed tomography
DEXA: Dual-energy X-ray absorptiometry
EWGSOP: The European Working Group on Sarcopenia in Older People
GH: Growth hormone
GLIS: The Global Leadership Initiative on Sarcopenia
IGF-1: Insulin-like growth factor 1
IL: Interleukin
MRI: Magnetic resonance imaging
mTOR: Mammalian target of rapamycin
p70S6K: Ribosomal protein S6 kinase
OR: Odds ratio
RCT: Randomized controlled trials
SARC-F: Strength, assistance in walking, rising from a chair, climbing stairs, and falls
SARC-CalF: SARC and calf circumference
SARMs: Selective androgen receptor modulators
SarQoL: Sarcopenia Quality of Life questionnaire
SPPB: Short physical performance battery
TKA: Total knee arthroplasty
TNF: Tumor necrosis factor
TUG: Timed-up and go-test
